# Immunolocalization of zinc transporters and metallothioneins reveals links to microvascular morphology and functions

**DOI:** 10.1007/s00418-022-02138-5

**Published:** 2022-07-18

**Authors:** Hai B. Tran, Rachel Jakobczak, Adrian Abdo, Patrick Asare, Paul Reynolds, John Beltrame, Sandra Hodge, Peter Zalewski

**Affiliations:** 1 Department of Thoracic Medicine, Royal Adelaide Hospital, School of Medicine, University of Adelaide, Adelaide, SA Australia; 2 Translational Vascular Function Research Collaborative, Basil Hetzel Institute for Translational Health Research and The Queen Elizabeth Hospital, University of Adelaide, Adelaide, SA Australia

**Keywords:** Vascular dysfunction, Pulmonary arterial hypertension, SLC39A/ZIPs zinc transporters, ZIP12, Metallothioneins, Multifluorescence quantitative confocal microscopy

## Abstract

Zinc homeostasis is vital to immune and other organ system functions, yet over a quarter of the world’s population is zinc deficient. Abnormal zinc transport or storage protein expression has been linked to diseases, such as cancer and chronic obstructive pulmonary disorder. Although recent studies indicate a role for zinc regulation in vascular functions and diseases, detailed knowledge of the mechanisms involved remains unknown. This study aimed to assess protein expression and localization of zinc transporters of the SLC39A/ZIP family (ZIPs) and metallothioneins (MTs) in human subcutaneous microvessels and to relate them to morphological features and expression of function-related molecules in the microvasculature. Microvessels in paraffin biopsies of subcutaneous adipose tissues from 14 patients undergoing hernia reconstruction surgery were analysed for 9 ZIPs and 3 MT proteins by MQCM (multifluorescence quantitative confocal microscopy). Zinc regulation proteins detected in human microvasculature included ZIP1, ZIP2, ZIP8, ZIP10, ZIP12, ZIP14 and MT1-3, which showed differential localization among endothelial and smooth muscle cells. ZIP1, ZIP2, ZIP12 and MT3 showed significantly (*p* < 0.05) increased immunoreactivities, in association with increased microvascular muscularization, and upregulated ET-1, α-SMA and the active form of p38 MAPK (Thr180/Tyr182 phosphorylated, p38 MAPK-P). These findings support roles of the zinc regulation system in microvascular physiology and diseases.

**Supplementary Information:**

The online version contains supplementary material available at 10.1007/s00418-022-02138-5.

## Introduction

The complex system of small blood vessels, namely arterioles, capillaries and venules, collectively called microvessels, is central in life-threatening conditions such as pulmonary arterial hypertension (PAH), coronary microvascular disease and microvascular brain disease. The microvascular wall consists of a few cell types, of which endothelial and smooth muscle cells are the two major populations in arterioles (diameter 10–100 µm) and venules (12–400 µm). In healthy adult microvessels, both these cell types are relatively quiescent, not proliferating, but sensitive to chemical or mechanical stimuli for activation (Ricard et al. 2021; Ourne et al. 2021). This activation may lead to increased cell proliferation and even switching of the smooth muscle cell phenotype (Ourne et al. 2021; Bkaily et al. 2021), resulting in a broad range of morphological and physiological changes known as “vascular remodelling” (Mulvany 1999). In animal models of diabetes and metabolic syndrome, hypertrophic remodelling of microvessels was identified in early stages when coronary arteries remained normal by angiography, indicating a key role for remodelling of micro- rather than macrovessels in initiation of haemodynamic disorders in these disease (Lambazi & Trask 2017). Multiple cellular and molecular events are ascribed to mechanisms of microvascular remodelling in pulmonary arterial hypertension, including proliferation and hypertrophy of smooth muscle cells, instigated in particular by signalling cascades with calcium (Masson et al. 2021), hypoxia-induced factor (Liu et al. 2019), sphingosine-1-phosphate (Ranasinghe et al. 2020), etc.

Zinc homeostasis is vital for functioning of the immune and other organs and systems. Cellular zinc homeostasis is regulated by three major families of proteins: (1) Solute Carrier 39 family/Zrt- and Irt-like proteins (SLC39A/ZIPs), which *import* zinc ions into the cytosolic compartment from the extracellular space or intracellular vesicles; (2) Solute Carrier 30 family/Zinc transporters (SLC30/ZnTs), which *export* zinc ions from the cytosolic compartment to the extracellular space, or intracellular vesicles; (3) metallothioneins (MTs), which have a high zinc binding capacity, thus playing key roles in intracellular zinc storage and buffering. To date, 14 ZIPs, 10 ZnTs and 4 isoforms of MTs with multiple subtypes/variants have been described in mammals (Kambe et al. 2021).

ZIPs have been identified as playing major roles in a broad array of vital functions and diseases (Takagishi et al. 2017). Their expression and functional roles in vascular physiology and diseases had been paid little attention (Zalewski et al. 2019) until the recent ground-breaking finding that ZIP12 is at least partly responsible for hypoxia-induced PAH in both human and rats (Zhao et al. 2015), inspiring other studies into this field (Tran et al. 2021; Xiao et al. 2021; Zhu et al. 2022). Data on vascular expression and functions of other members of the zinc regulation system remain scant. ZIP14 was shown to mediate influx of Zn^2+^ in sheep pulmonary artery endothelial cells, which may act together with MT to protect against LPS-induced apoptosis (Thambiayya et al., 2012). MT expression and anti-oxidative stress functions in vasculature have been implicated in a number of studies using models of cultured endothelial cells (Kaji et al. 1993; Conway et al. 2010; Thambiayya et al. 2012; Fujie et al. 2020). Thus, despite a growing interest into the zinc regulation system in vascular health and diseases, the understanding of vascular expression and functions of ZIPs, ZnTs and MTs in vivo remains a large gap in our knowledge. A systematic background analysis of the zinc regulation system in human vasculature would benefit further investigations in this field.

Following on from our previous study (Abdo et al. 2021) aiming to characterize the zinc regulation system in human vasculature, in this study we employed multifluorescence quantitative confocal microscopy (MQCM) to investigate immunoreactivities of human microvessels in paraffin tissue sections for multiple ZIPs and MTs. Their detailed distribution among the major cell types of microvessels and association with microvascular morphology and expression of vascular function-related molecules was investigated.

## Materials and methods

### Antibodies

A panel of 13 primary antibodies to zinc transporters and metallothioneins and 6 to other molecules was used in this study. Their source, animal species, class/type, dilutions, immunogens and published details relating to specificity are summarized in Supporting Information Table S1.

To minimize cross reactivity in multifluorescence labelling, all secondary antibodies were donkey IgG F(ab’)2 fragments, absorbed against cross-species reactivities. They were obtained from Jackson ImmunoResearch, including anti-rabbit IgG-AF594, anti-goat IgG-AF488 and anti-mouse IgG-AF647, used at 1:200. For alternative combinations of primary antibodies in some experiments conjugates switched colours (anti-rabbit IgG-AF488, anti-goat IgG-AF594, anti-goat IgG-AF647 and anti-mouse IgG-AF488).

All primary and secondary antibodies were diluted in PBS with 0.5% Tween-20 and 10% serum-free protein blocker (DakoCytomation Inc., Carpinteria, CA, USA) added.

### Human tissue samples

Subcutaneous tissue biopsies were collected from 14 patients undergoing hernia reconstructive surgeries at The Queen Elizabeth Hospital in Adelaide, Australia. Informed consent was obtained from each donor, utilising protocols approved by the Central Adelaide Local Health Network Human Research Ethics Committee at The Queen Elizabeth Hospital (approval number 2009012). According to the ethics approval, individual patient demographic data and their disease status were known only to three authors, RJ, JB and PZ.

### Tissue processing and histology

To minimize variations that could have resulted from tissue processing, the pre-fixation time when tissue samples were preserved in RPMI medium on ice for transport was kept < 2 h; the fixation protocol was kept uniformly for 24 h at room temperature in phosphate saline-buffered 10% formalin, a protocol accepted by most authors (Engel and Moore 2011). For quantitative analysis, sections from multiple paraffin blocks were cut to 5 µm thickness and mounted onto tissue arrays for batch analysis of all samples.

Tissue sections were stained with H&E in a standard protocol at the Histopathology service at Adelaide Health and Medical Sciences and then scanned at a 40× objective with a Nanozoomer digital slide scanner (Hamamatsu Photonics, Hamamatsu, Shizuoka, Japan).

### Multifluorescence quantitative confocal microscopy (MQCM)

Immunofluorescence of human paraffin tissue sections was performed following a protocol described in our previous study (Tran et al. 2021). MQCM was carried out using an Olympus confocal microscopy system (Olympus FV3000, Tokyo, Japan) and ImageJ morphometric software (NIH, MA, USA) as previously described (Tran et al. 2021). Briefly, ten optical fields containing microvessels were serially captured from each biopsy under a 60× silicone-immersed objective simultaneously in four fluorescence channels set for AF488, AF594, AF647 and DAPI. All microvessels of 20–100 µm diameter in each frame were then selected and their areas in monochromatic images measured for mean fluorescence intensities (MFI) by ImageJ. Analogous to the flow cytometry mean fluorescence intensity (MFI), which is a quantitative measurement of fluorescence brightness averaged from all events counted in a gate, in immunofluorescence MFI is averaged from all pixels of the region of interest (ROI) of an image. MFI values were measured using MQCM as follows. From a merged multi-colour confocal image, the area of a microvessel section as the ROI was delineated first using the ImageJ software drawing tools. Using the CTR-SHIFT-E keys, the ROI was then applied to monochromatic channel images which had previously been converted to greyscale by menu Image/Type/16-bit. Then, the menu Image/Adjust/Threshold was applied, allowing the software to automatically predict a threshold. Next, in the menu Analyze/Analyze Particles, the mean greyvalue was selected from Set Measurement to measure the ROI MFI. The MFIs of ten ROIs captured from each sample were averaged and corrected for autofluorescent background. The latter were measured for each channel in a similar way as for the samples stained with conjugate alone (negative controls, which were included in every batch analysis). For punctate immunofluorescence of ET-1, particle counting function was carried out in a predetermined threshold band (70, with maximum intensities varying between 44 and 154 in the AF488 channel) for uniform gating in only bright particle sizing of > 10 square pixels (> 3.13 µm^2^). Numbers of particles counted in a vascular area were then normalized by numbers of nuclei counted in the DAPI channel in the same area.

Microvessels were subdivided into two subpopulations, ‘muscularized’ when having walls consisting of at least two cell layers in the whole perimeter and ‘non-muscularized’ for walls consisting of fewer than two layers. The percentage of ‘muscularized’ microvessels varied between 0 and ~ 60%.

After obtaining quantitative results of all included samples, representative confocal images were selected for those most closely reflecting the final quantitative results, without referring to the donor age and disease status.

### Statistical analysis

Statistical analysis was undertaken using Prism 9 software (GraphPad Software, CA, USA). For difference between subgroups of microvessels, a paired two-tailed Wilcoxon test was used. Changes were considered statistically significant at *p* < 0.05.

## Results

### Patient characteristics

Tissues donated from 14 patients (10 males, age range: 23–91 years) undergoing hernia reconstructive surgeries were analysed. Their demographic characteristics, body/mass index, usage of vitamin or zinc supplements, cigarette smoking status and history of pathological conditions known at the time of surgeries are summarized in Supporting Information Table S1. Cardiovascular diseases were reported in four cases (infarct, 1; deep thrombosis of lower extremity, 1). Conditions that may be risk factors for cardiovascular diseases included hypertension (2), diabetes (3), asthma (2) and COPD (1). No history was reported for four.

### Morphology of human subcutaneous microvessels

**Fig. 1:**
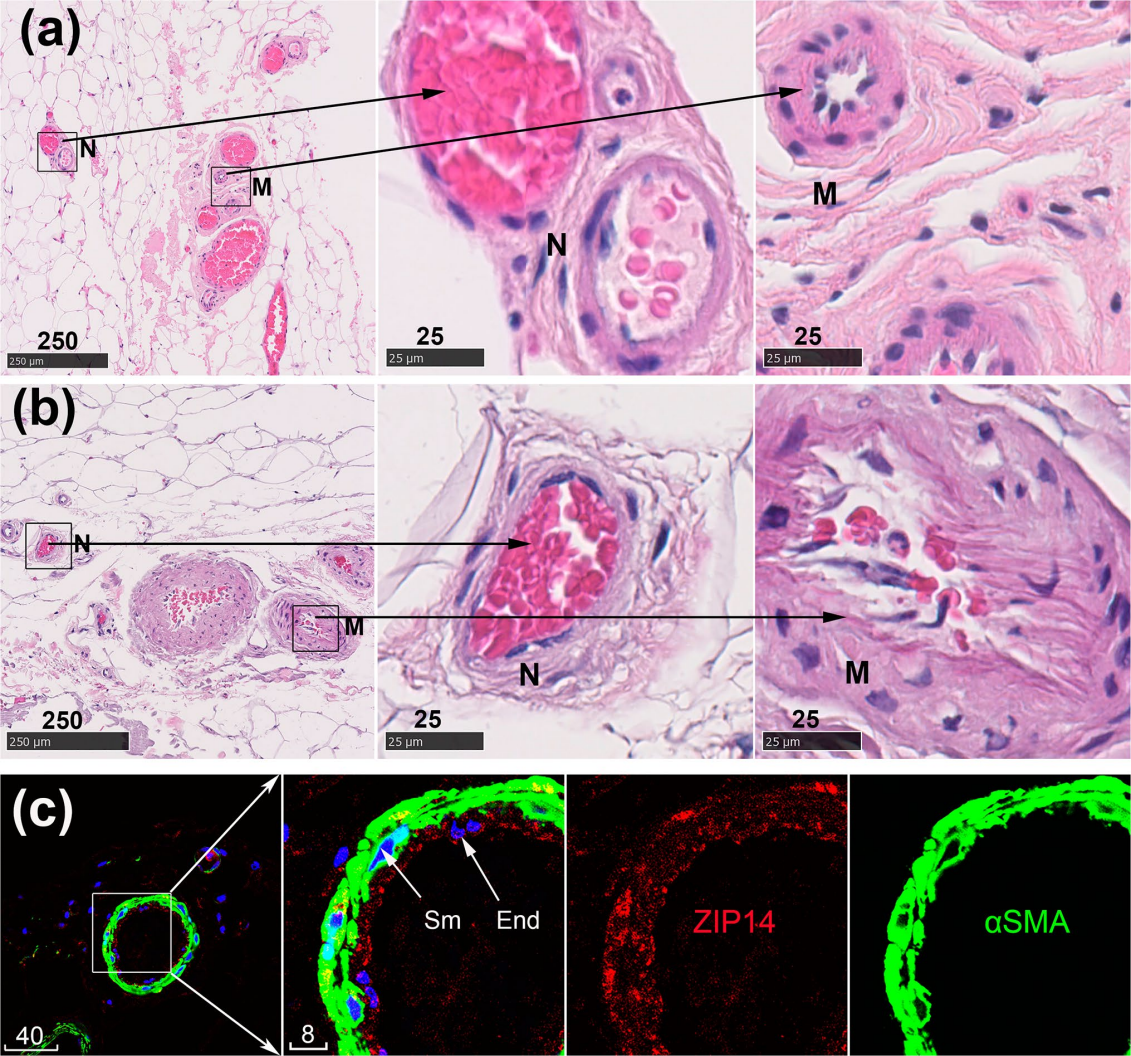
Morphology of human subcutaneous microvessels. **a** and **b** Representative H&E microphotos of microvessels in biopsies of two different donors. Boxed areas are shown to the right at a higher magnification to reveal relatively non-muscularized (N) and muscularized (M) microvessels. **c** A representative multifluorescence confocal image of subcutaneous tissue stained for ZIP14 (red), α-SMA (green) and nuclei (blue, DAPI). The boxed area is shown to the right at a higher magnification, revealing different layers of the microvascular wall: Sm, smooth muscle; End, endothelium. Scale bars are in micrometres

In H&E staining subcutaneous biopsies consisted of mostly adipocytes, scattered with islands of dense irregular connective tissue, nerve fibres and microvessels. By applying the inclusion criterion of diameter between 20 to 100 µm, the selected microvessels included both venules and arterioles but excluded much smaller capillaries (< 10 µm by definition). The microvessels varied in their wall thickness and relative degree of muscularization (Fig. 1). Muscularized microvessels showed increased wall-to-lumen projection, often having rough endothelial surfaces, increased nuclear density and fibrotic staining in the tunica intima (Fig. 1a, b).

At high-resolution confocal microscopy, microvascular cell layers of tunica intima (endothelium) and tunica media (smooth muscle) could be clearly demarcated, allowing for sub-classification by degree of muscularization (Fig. 1c).

### Localization of multiple ZIPs and MTs in human microvasculature

**Fig. 2:**
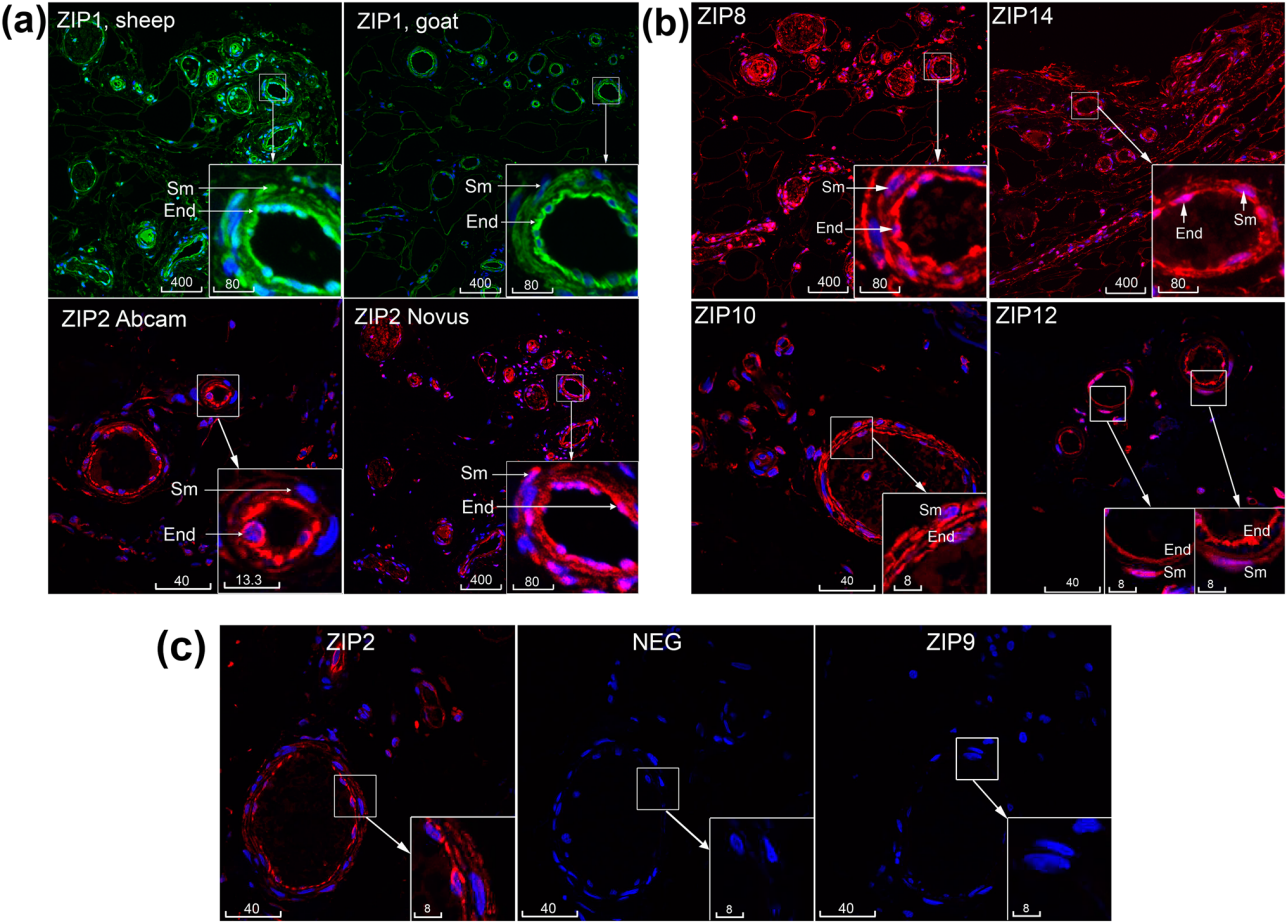
Immunolocalization of multiple ZIPs in microvessels. **a** Comparison of ZIP1 (green) and ZIP2 (red) antibodies from different sources. **b** Immunolocalization of ZIP8, ZIP10, ZIP14 and ZIP12 (all red) in microvessels. **c** Positive vs. negative staining. In the same batch experiment, three serial sections of the same microvessel gave weak but positive staining of ZIP2 (red, AF594) compared to negative ZIP9 and the conjugate alone negative control (NEG). Blue is DAPI. Sm, smooth muscle; End, endothelium. Scale bars are in micrometres

**Fig. 3:**
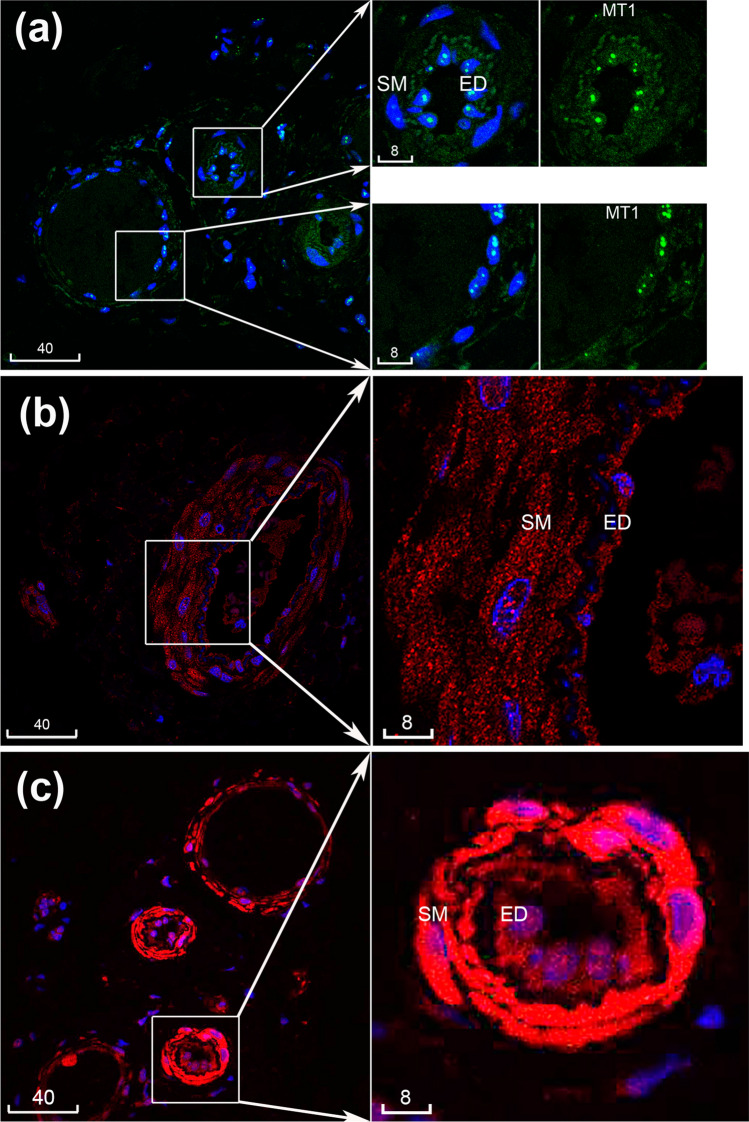
Immunolocalization of multiple metallothioneins in microvessels. **a** MT1 (green), **b** MT1/2 (red), **c** MT3 (red). Blue is DAPI. Sm, smooth muscle; End, endothelium. Scale bars are in micrometres

Preliminary experiments were carried out to titrate primary antibodies and define optimal dilutions, as described in the Methods. In the conditions of our protocols, similar patterns of ZIP1 immunofluorescence in the endothelium and smooth muscle were detected by a homemade sheep antibody (Michalczyk and Ackland 2013) and a commercial goat antibody (Fig. 2a). For ZIP2, similar patterns of endothelium and smooth muscle staining were detected using the two rabbit polyclonal antibodies (Abcam and Novus, Fig. 2a). Other ZIPs detected in microvessels were ZIP8, ZIP10, ZIP12 and ZIP14 (Figs. 1b, 2b). In the described protocol the Abcam antibodies to ZIP6 and ZIP9 did not give immunofluorescence exceeding the level of background fluorescence in the vasculature (Fig. 2c). Next, all three tested antibodies to MTs revealed positive immunoreactivities in human microvessels (Fig. 3).

High resolution by confocal microscopy allowed detailed localization of ZIPs and MTs, roughly equally in both endothelium and smooth muscle (ZIP10, ZIP14, MT1, MT1/2) or relatively more abundantly in endothelium (ZIP1, ZIP2, ZIP8, ZIP12) or smooth muscle (MT3) (Figs. 1b, 2, 3). Immunofluorescence of ZIPs showed both diffused cytoplasmic and thin cytoplasmic patterns; the latter was particularly distinctive for ZIP1 and ZIP12. Immunofluorescence of MT1/2 and MT1 with monoclonal antibodies detected bright spots overlapping the nucleus and weaker cytoplasmic staining in both endothelium and smooth muscle. MT3 immunofluorescence with a polyclonal antibody revealed bright cytoplasmic staining in smooth muscle and weaker staining in endothelium (Fig. 3).

### Changes of ZIP and MT immunoreactivities associated with microvascular muscularization

**Fig. 4:**
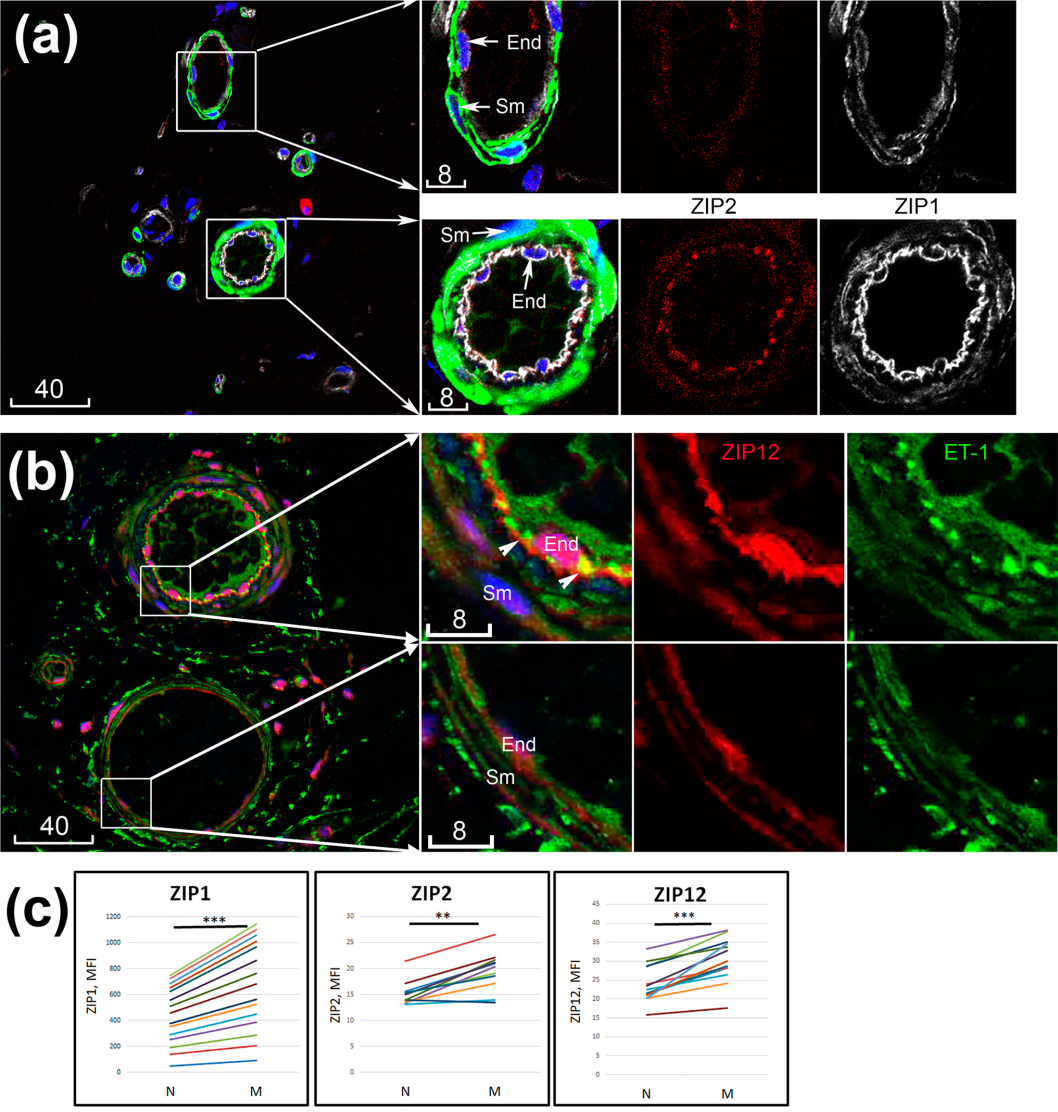
Upregulated immunoreactivities of ZIPs in muscularized vs. non-muscularized microvascular walls. **a** Confocal image of ZIP1 (white) and ZIP2 (red) co-labelled with α-SMA (green); two boxed areas shown to the right at higher magnification reveal a relatively non-muscularized (top) vs. muscularized (bottom) microvessel. **b** Confocal image of ZIP12 (red) co-labelled with ET-1 (green, arrowheads point to endothelial-smooth muscle junctions); the two boxed areas shown to the right at higher magnification reveal a relatively muscularized (top) vs. non-muscularized (bottom) microvessel. Blue is DAPI counterstaining of nuclei. Sm, smooth muscle; End, endothelium. Scale bars are in micrometres. **c** Quantitative measurements of ZIP1, ZIP2 and ZIP12 in non-muscularized (N) vs. muscularized (M) microvessels. **p = 0.01; ***p < 0.001

**Fig. 5:**
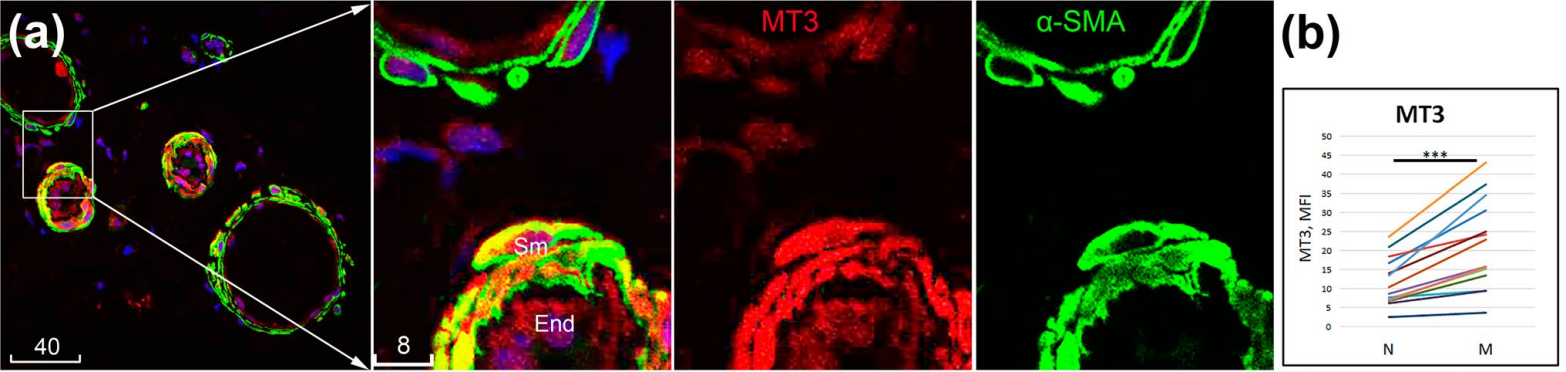
Upregulated immunoreactivity of MT3 in muscularized microvessels. **a** Representative confocal image of MT3 (red) co-labelled with α-SMA (green). The boxed area is shown to the right at a higher magnification to reveal a relatively non-muscularized (top) and muscularized (bottom) microvessel. Blue is DAPI. Sm, smooth muscle; End, endothelium. Scale bars are in micrometres. **b** Quantitative measurements of MT3 immunoreactivities in non-muscularized (N) vs. muscularized (M) microvascular walls. ***p < 0.001

The limited number of patients and heterogeneity of their history would not allow for a quantitative analysis of potential changes of ZIP/MT immunoreactivities by disease or by age/gender subgroups. However, a distinctive variation of ZIP1, ZIP2, ZIP12 and MT3 among microvessels of the same donor led us to postulate that there are changes associated with microvascular morphology. For the purpose of quantitative analysis, the relatively “muscularized” were discerned from “non-muscularized” microvessels, arbitrarily based on whether their wall contained at least two muscle cell layers throughout their perimeters. The microvascular immunoreactivities of ZIP1, ZIP2, ZIP12 and MT3 quantified by their mean fluorescence intensities confirmed statistically significant increases in muscularized vs. non-muscularized microvessels (Figs. 4, 5). Of note, while upregulated ZIP1, ZIP2 and ZIP12 were recorded mostly in the endothelium, increased MT3 was mostly seen in the smooth muscle compartment.

### Change in microvascular muscularization was associated with vascular function-related molecules

**Fig. 6:**
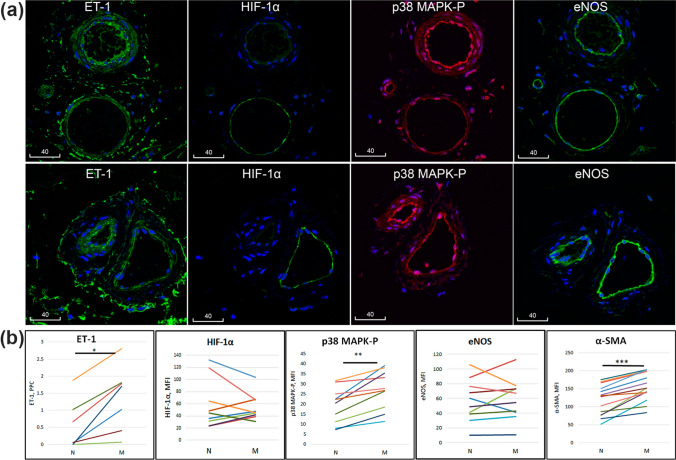
Immunolocalization of function-related proteins and their variation among microvessels of the same donors. **a** Confocal images of adjacent serial sections of microvessels from two representative donors, stained for various function-related molecules. Blue is pseudocolour of DAPI. Scale bars are in micrometres. **b** Quantitation of immunoreactivities in non-muscularized (N) vs. muscularized (M) microvessels. *p < 0.05; **p < 0.01; ***p < 0.001

Next, we tested whether muscularized and non-muscularized subpopulations of microvessels would display any difference regarding expression and localization of molecules that have previously recognized roles in vascular functions. A panel of function-related markers was investigated including eNOS, ET-1, HIF-1α, α-SMA and p38 MAPK. As expected, while the α-SMA immunoreactivity was localized specifically to the smooth muscle (Fig. 5a), most of ET-1 (Fig. 4b) and eNOS (Fig. 6a) were localized to the endothelium. Notably, punctate ET-1 immunofluorescence could be visualized in endothelial-smooth muscle junctions on the endothelial side (Fig. 4b). HIF-1α was mostly localized to the endothelium (Fig. 6a). Interestingly, while staining with an antibody to the total p38 MAPK detected bright immunoreactivity in both smooth muscle and endothelium, the active form of p38 MAPK-P was mostly localized to the latter (Supporting Information Figure S1).

Importantly, in quantitative analysis ET-1, p38 MAPK-P and α-SMA revealed significant increases in muscularized microvessels. Although eNOS and HIF-α also showed a strong variation among microvessels of the same donor, their overall change among all donors was not statistically significant, increasing with muscularization in some donors but decreasing in others (Fig. 6b).

## Discussion

To our best knowledge this work is the first in vivo description of protein expression of multiple ZIPs and MTs in human microvasculature. A complete list analysis of the ZIPs and MTs and the third family of zinc regulation proteins, SLC30As/ZnTs, remains however a task for future investigations. Despite a limitation that expression at the gene level was not analysed, protein expression of multiple members of ZIPs and MTs supports the hypothesis that there is a redundancy in the zinc regulation system which may be required for a tight control of zinc homeostasis in vascular functions. As another limitation, immunofluorescence results should be interpreted with caution in relation to antibodies’ specificity. To minimize this, most of the primary antibodies used in this study were tested independently by Western blots or immunogen/antibody competition in our previous publications, or published by the manufacturers; furthermore, fragment secondary antibodies pre-absorbed against cross-species reactivities were employed.

Results of this study are mostly in line with our previous data on gene expression in primary cell culture (Abdo et al. 2021), with exceptions as discussed below. Thus, while abundant mRNA expression was found in primary cell cultures for ZIP6 and ZIP9, their protein expression in this study could not be detected in microvessels from subcutaneous biopsies. Furthermore, while the relative abundance of gene expression in cell cultures was low for ZIP2, ZIP12 and MT3, protein immunoreactivities in microvascular tissues were varying and brighter in subpopulation(s) of microvessels. Apart from a potential sensitivity issue that could not be ruled out, one possible cause was that while mRNA data were obtained from primary cells of aorta and pulmonary artery, protein expression data were from in vivo sampling of microvessels. Furthermore, while primary cell culture data reflected a normal state in which vascular cells expressed minimal levels of ZIP2, ZIP12 and MT3, biopsies included pathological conditions that could induce these proteins. In accordance with this, previous data provide multiple evidence that ZIP12 could be induced in vivo in vasculature in human patients and rat models of PAH (Zhao et al. 2015; Tran et al. 2021; Xiao et al. 2021). In vitro, both ZIP2 and ZIP12 were shown to be induced at mRNA and protein levels in vascular cells by depletion of zinc (Abdo et al. 2021). The tissue localization data in this study were in line with previous studies (Zhao et al. 2015; Abdo et al. 2021; Tran et al. 2021); ZIP12 was localized to both the endothelial and smooth muscle cell types of the vascular wall. Microvascular expression of ZIP14 was consistent with previous data in that influx of labile zinc in cultured sheep pulmonary artery endothelial cells was sensitive to ZIP14 siRNA indicating the presence of functional ZIP14 in this cell type (Thambiayya et al. 2012). Our findings of highly expressed ZIP1 in microvessels were in line with a notion that ZIP1 is expressed ubiquitously across cell types (Schweigel-Röntgen 2014). Regarding other ZIPs, to our knowledge ours is the first study to examine their localization in vascular walls.

Known as free radical scavengers, MTs are surrogate markers of oxidative stress and indicators of labile intracellular zinc levels (Mareiro et al. 2017). MTs have been studied in various cell culture models of vascular endothelial cells in oxidative stress associated with exposure to heavy metals or other stress stimuli (Kaji et al. 1993; Conway et al. 2010; Thambiayya et al. 2012; Fujie et al. 2020; Rubiolo et al. 2021). Metallothioneins have also been commonly reported to be elevated in PAH patients as well as in experimental models of PAH (Maarman 2018). In vitro studies showed that MT can respond to nitride oxide (free radical and vasodilator mediator) by releasing Zn2 + ions (Kroncke et al. 1994; Thambiayya et al. 2012), which could be relevant to a mechanism of vasodilation. Surprisingly MT expression and functions in the other vascular cell type, namely smooth muscle, have been paid little attention. In a rare report, using immunohistochemical staining and immunogold electron microscopy, it was noted that most of the MT induced in human atherosclerotic lesions was localized to the vascular smooth muscle cells (Göbel et al. 2000). Our finding of smooth muscle as the major harbour of MTs in the microvascular wall further puts this cell type in the spotlight of future investigations into vascular zinc biology.

By the selected projection size (20−100 µm), microvessels analysed in this study fall into categories of arterioles and venules, which can be roughly distinguished from each other histologically by the level of their muscularization and wall to lumen ratio. A precise differentiation between arterioles and venules requires their localization prior to or after capillary circulation by serial sections (Bonner-Weir and Orci 1982), or at least according to more detailed morphological characteristics, for example, by transmission electron microscopy (Sharp et al 2019) or photoacoustic imaging (Matsumoto et al. 2018), which were not available within this study. Increased proliferation of smooth muscle layer is considered a key process in vascular pathology, for example, in atherosclerosis (Sedding et al. 2018) and diabetic vascular restenosis (Moshapa et al. 2019). In studies of PAH, increased levels of lung microvascular muscularization serve as an indicator of pathological changes leading to resistance and increased pulmonary blood pressure (Zhao et al. 2015; Harper et al. 2019; Maietta et al. 2021). The data presented here indicate that the level of ZIP1, ZIP2, ZIP12 and MT3 expression is higher in those microvessels having increased muscularity, which are likely to be arterioles rather than venules. However, this needs to be confirmed in further studies.

The differences between the two microvessel subpopulations in their expression of vascular-active molecules, however, give a notable indication on functional differentiation, in addition to morphological features. Known primarily as a potent vasoconstrictor, ET-1 has broad effects on various pathways critical for vascular functions and diseases, for example, induction of VCAM-1 (Ishizuka 1999), pro-inflammatory activation of leucocytes including neutrophils (Kaszaki et al. 2008) and macrophages (Zhang et al. 2021). Relevant to the vascular pathology, ET-1 was reported to activate smooth muscle by stimulating protein synthesis, promoting proliferation and hypertrophy of pulmonary arterial smooth muscle cells (Chua et al. 1992; Zamora et al. 1993). In this study increased particulate immunofluorescence of ET-1 in muscularized microvessels was localized to endothelial-smooth muscle junctions, supporting the hypothesis that paracrine ET-1-mediated endothelial-smooth muscle crosstalk may be required for not only vasoconstriction, but also proliferation of vascular smooth muscle. As a marker of both endothelial-mesenchymal transition and vascular remodelling, increased α-SMA expression is known to be associated with exposure to mechanical stress, which could also induce activation of p38 MAPK (Wang et al. 2006). In a study of mechanisms leading to PAH, activation of p38 MAPK was found to be associated with oxidative stress and inflammation (Church et al. 2015). Thus, data presented here argue that upregulation of zinc regulation proteins ZIP1, ZIP2, ZIP2 and MT3 is associated with a functionally activated state compared to a relatively quiescent state of microvessels.

## Conclusion

In conclusion, this study provides background data of protein expression and localization of multiple ZIPs and MTs in endothelial and smooth muscle layers of human microvascular walls. The presented data support the hypothesis that the zinc regulation system in the human microvasculature, in particular the ZIP1, ZIP2, ZIP12 and MT3 proteins, plays an important role in microvascular physiology and could be a therapeutic target for diseases that involve microvascular remodelling.

## Supplementary Information

Below is the link to the electronic supplementary material.

Supplementary file1 (PDF 3251 KB)

## Data Availability

All the data supporting the findings of this study are available within the article and its supplementary materials.
