# Supplementary material for: Immunolocalization of zinc transporters and metallothioneins reveals links to microvascular morphology and functions
Source: Histochem Cell Biol. 2022 Jul 18;158(5):485–96. doi: 10.1007/s00418-022-02138-5 (PMC9630201; doi:10.1007/s00418-022-02138-5)
Supplement: Supplementary file 1 — Supplementary file1 (PDF 3251 KB) [file 418_2022_2138_MOESM1_ESM.pdf]

Supporting Information

Tran et al

Table S1. Primary antibodies

Antibodies	Source	Species	Type	Dilution	Immunogen	Note
ZIP1	Home made	Sheep	pAb	1:100	N-terminal sequence	Specificity confirmed by immunoblots and antigen/antibody competition (Michalczyk & Ackland, 2013)
ZIP1	Santa Cruz	Goat	pAb	1:50	Cytoplasmic sequence of mouse ZIP1	Affinity purified
ZIP2	Abcam	Rabbit	pAb	1:50	Intracellular sequence	Immunoblot shown by the manufacturer
ZIP2	Novus	Rabbit	pAb	1:20	Intracellular sequence	Immunoblot shown by the manufacturer
ZIP6	Abcam	Rabbit	mAb recombinant	1:25	Immunogen undisclosed	Immunoblot shown by the manufacturer
ZIP8	Sigma-Aldrich	Rabbit	pAb	1:50	Epitope undisclosed	Affinity purified, immunoblot shown by the manufacturer
ZIP9	Abcam	Rabbit	pAb	1:30	Internal sequence	Immunogen affinity purified
ZIP10	Abcam	Rabbit	pAb	1:100	N-terminal sequence	Immunogen affinity purified
ZIP12	Abcam	Rabbit	pAb	1:50	N-terminal sequence	Immunoblot published Tran et al 2021
ZIP14	Abcam	Rabbit	pAb	1:50	Internal sequence	Immunogen affinity purified
MT3	Sigma-Aldrich	Rabbit	pAb	1:50	Immunogen undisclosed	Affinity purified, RNAseq confirmation on tissue sections
MT1 Clone UC1MT	Abcam	Mouse	mAb IgG1	1:100	Immunogen was a full length protein	Immunoblot shown by the manufacturer, 50 plus references
MT1/2 Clone E9	Dako	Mouse	mAb IgG1	1:40	Immunogen undisclosed	Immunoblot shown by the manufacturer
ET-1	Santa Cruz	Goat	pAb	1:50	Internal sequence	Immunoblot shown by the manufacturer
eNOS Clone 3/eNOS	BD	Mouse	mAb IgG1	1:50	Aa2025-1203 sequence	Immunoblot shown by the manufacturer
p38 MAPK	Cell Signalling	Rabbit	pAb	1:50	Peptide sequence undisclosed	Immunogen affinity purified Immunoblots published Harper et al 2019
p38 MAPK, phosphorylation	Cell Signalling	Rabbit	pAb	1:50	Phosphopeptide including Thr180/Tyr182	Immunogen affinity purified Immunoblots published Harper et al 2019
HIF-1α Clone 54/HIF-1a	BD	Mouse	mAb IgG1	1:20	Aa610-727	Knocked out validated specificity
α-SMA Clone 1A4	Dako	Mouse	mAb IgG2a	1:200	N-terminal synthetic decapeptide	Immunoblots published, 40 plus references

Table S2. List of patients

ID #	Gend er	Age (years)	BMI	Vitamin or Zn	Veget arian	Beans	Red meat		Sea/shellfood				Smoker	cigs/day	Alcohol			Questionaire	Patient History
				suppltss		Daily	Often	Sometimes	Often	metim	Never	Unknown			Often	Sometimes	Never		
1	Male	62	21.9															×	Asthma
2	Male	23	25.6	×	×	×	.	✓	.	.	✓	.	×	.	.	.	✓	✓	Nil History
3	Female	78	34.1	×	×	×	.	✓	.	✓	.	.	×	.	✓	.	.	✓	COPD, Asthma, Obstructive sleep apnoea, Chronic depression, Diverticular disease, Deep venous thrombosis of lower extremity, Diabetes mellitus type 2, Reflux, Atrial fibrillation.
4	Male	49	27.7	×	×	×	.	✓	.	.	.	.	×	.	✓	.	.	✓	Nil History
5	Female	63	29.7	✓	×	×	.	✓	.	✓	.	.	×	.	.	✓	.	✓	Depressive disorder, Schizophrenic, Bilateral breast surgery
6	Male	75	32.8	×	×	✓	.	✓	.	✓	.	.	×	.	.	.	✓	✓	Nil History
7	Male	64	25.2	×	×	×	✓	.	.	✓	.	.	×	.	✓	.	.	✓	Nil History
8	Male	40	37.9	✓	×	✓	.	✓	.	.	✓	.	✓	5	.	✓	.	✓	Diabetes, GORD
9	Female	67	43.3	×	×	✓	✓	.	.	✓	.	.	×	.	.	✓	.	✓	SOBOE, Asthma, Morbid obesity, Diabetes mellitus type 2, Hyperlipidaemia, Essential hypertension, Rheumatoid arthritis, Obstructive sleep apnoea syndrome - good compliance w CPAP
10	Male	75	25.3	×	×	×	.	✓	.	✓	.	.	×	.	.	✓	.	✓	STEMI 1980, old infarct, prev CABG, recent cardiac investigations
11	Male	91	23.9	×	×	×	✓	.	.	✓	.	.	×	.	.	.	×	✓	Hypertension, amputee
12	Female	39	25.3										×					×	Gastrooesophageal reflux disease. Hypothyroidism due to Hashimoto's thyroiditis. Anxiety. Palpitations. Ex-smoker
13	Male	36	31.3	×	×	×	✓	.	.	.	.	✓	×	.	.	✓	.	✓	Gout
14	Male	47	52.7										×					×	Cellulitis, Chronic depression, Asthma, Lymphoedema., Diabetes mellitus type 2. Sleep apnoea. Ex-smoker

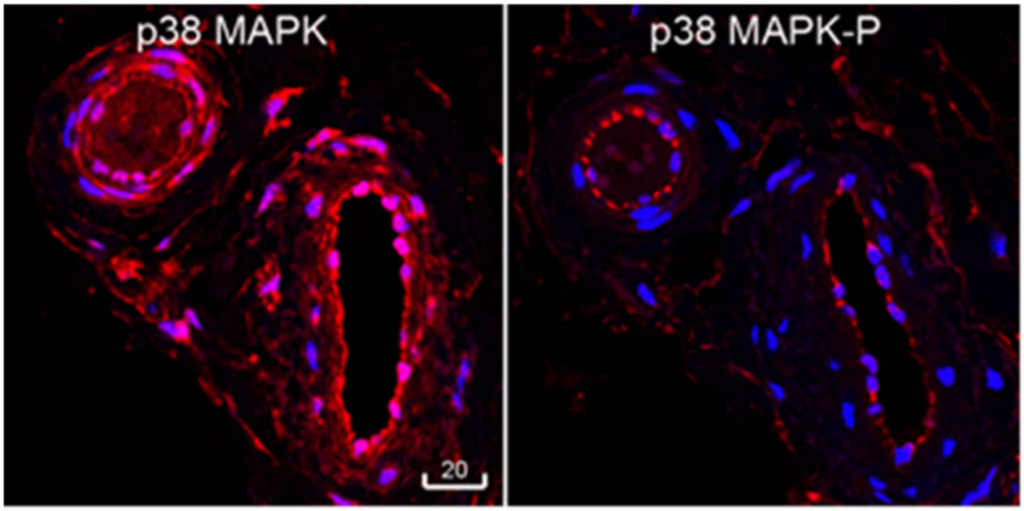

Figure S1. Immunolocalization of p38 MAPK (total, left) and its phosphorylated form (right) in serial sections of human subcutaneous microvessels. Red, immunofluorescence in AF594; blue, DAPI. Scale bar is in micrometers and applied for both images.

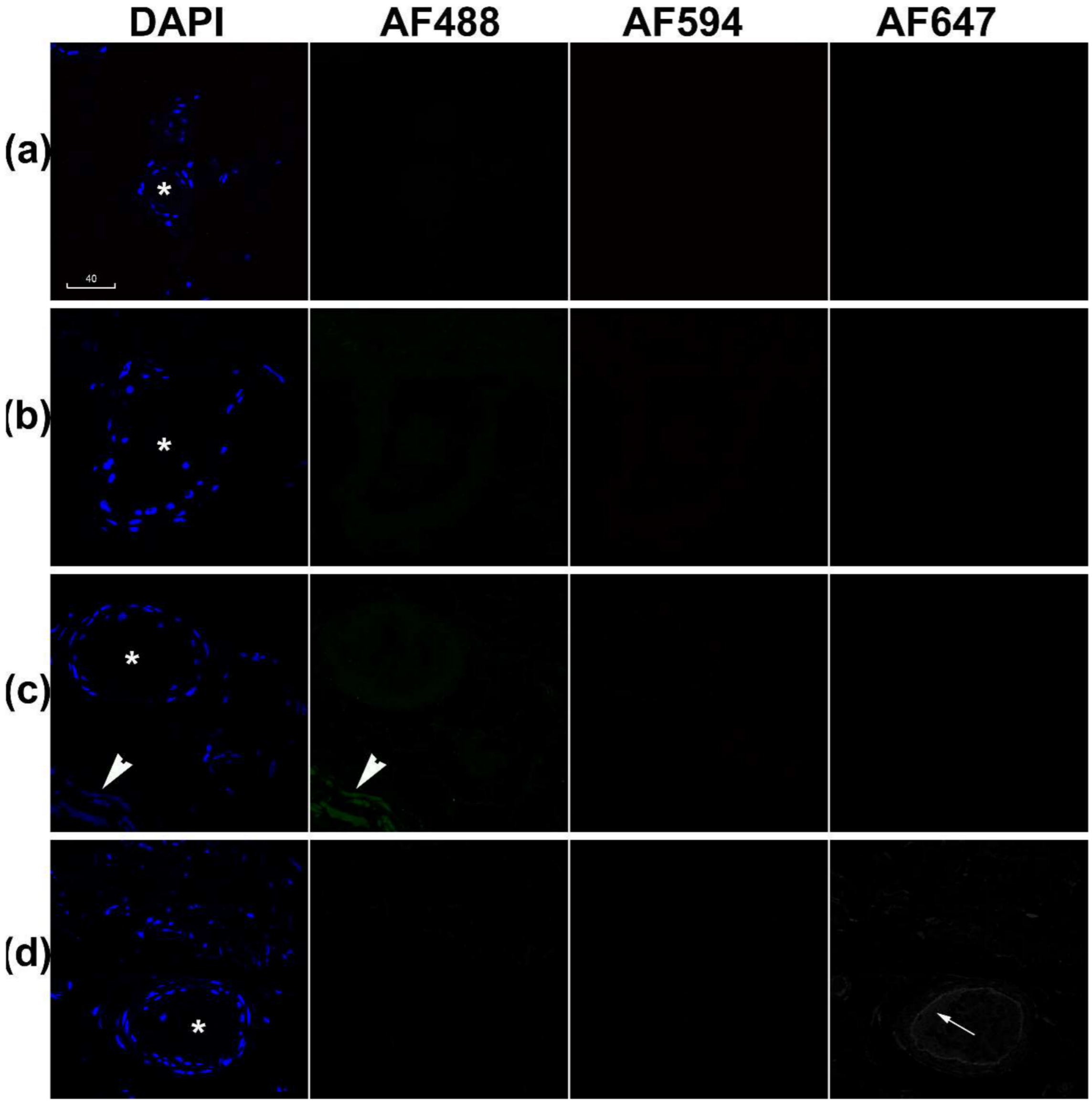

Figure S2. Representative confocal images of conjugate alone negative controls, employed for correction of quantitative measurement of MFI. Increased nonspecific fluorescence was recorded in large bundles of collagen fibres (arrowheads), the lumen and endothelial surface (short arrow). Microvessels recognizable in the DAPI channel are indicated with arterisks. Other channels are AF488 (green), AF594 (red), AF647 (far red, pseudocolored in white). Scale bar of 40 micrometers in (a) is applied for the whole pannel.
